# Case Report: Sphincter-preserving surgery after conversion therapy and longitudinal recovery of bowel function in ultra-low rectal cancer

**DOI:** 10.3389/fonc.2026.1957728

**Published:** 2026-09-09

**Authors:** Ruolin Fang, Chenxi Hu, Meimei Ren, Jinchang Huang, Ming Yang

**Affiliations:** 1 The Third Affiliated Hospital, Beijing University of Chinese Medicine, Beijing, China; 2 The Second Affiliated Hospital, Shaanxi University of Chinese Medicine, Xianyang, China; 3 Department of Acupuncture and Mini-invasive Oncology, Beijing University of Chinese Medicine Third Affiliated Hospital, Beijing, China

**Keywords:** Baliao points, electroacupuncture, low anterior resection syndrome, rectal cancer, sphincter-preserving

## Abstract

**Background:**

KRAS-mutated ultra-low rectal cancer is associated with poor response to conventional neoadjuvant therapy, making sphincter preservation particularly challenging. In addition, effective rehabilitation strategies for severe low anterior resection syndrome (LARS) remain limited.

**Case presentation:**

A 55-year-old woman with KRAS-mutated ultra-low rectal adenocarcinoma experienced insufficient tumor regression after multiple lines of chemotherapy, chemoradiotherapy, and immunotherapy, complicated by oxaliplatin allergy. After referral to our center, she received bevacizumab plus XELIRI combined with traditional Chinese medicine therapies. Following five treatment cycles, the patient underwent laparoscopic radical rectal resection with transanal specimen extraction and Bacon-type pull-through reconstruction. Pathological examination demonstrated negative proximal, distal, and circumferential margins, no metastatic involvement among 12 examined lymph nodes, and a final pathological stage of ypT2N0. The patient subsequently developed major LARS and underwent a four-week rehabilitation program centered on electroacupuncture at the Baliao acupoints. Assessment was conducted using the LARS score, Memorial Sloan Kettering Cancer Center Bowel Function Instrument (MSKCC-BFI), and EORTC QLQ-C30 scales. The patient’s bowel movement frequency decreased from over 30 times per day to approximately 10 times, and remained stable at 7–10 times during the 6-month follow-up.

**Conclusion:**

In this patient, an individualized conversion strategy was followed by R0 sphincter-preserving resection, and postoperative bowel function improved temporally during and after multimodal rehabilitation that included electroacupuncture. This observation provide preliminary evidence in support of this treatment approach. However, high-quality studies are still required to comprehensively evaluate the efficacy of this treatment regimen.

## Introduction

Colorectal cancer ranks as the third most common malignant tumor worldwide, with approximately 70% of cases occurring in the middle and lower rectum (1). The treatment of low rectal cancer (LRC) is particularly complex. Achieving radical resection with preservation of anal function through surgical intervention represents both the primary clinical goal for patients and a significant technical challenge (2). Cases where the tumor margin is less than 1 cm from the anal verge are typically classified as ultra-low rectal cancer (ULRC), for which anal-preserving surgery poses significant challenges and often requires abdominoperineal resection (APR) (3). APR not only carries a high recurrence rate but may also lead to permanent stoma formation. For locally advanced LRC, the current standard treatment regimen involves neoadjuvant therapy combined with total mesorectal excision (TME) (4). However, there is considerable individual variability in response to neoadjuvant therapy, with complete clinical response rates ranging only from 5% to 30% (5). Patients with normal mismatch repair (pMMR) or microsatellite stable (MSS) phenotypes exhibit limited response to single-dose immunotherapy (6). Patients with KRAS mutations exhibit primary resistance to targeted therapies and may represent a risk factor for anastomotic leakage (AL) following resection of the middle and lower rectum, thereby losing the opportunity for sphincter preservation (7, 8). Therefore, developing personalized treatment strategies for LRC patients with KRAS mutations and pMMR/MSS phenotypes, while balancing therapeutic efficacy with functional preservation, remains an unresolved clinical challenge.

Although sphincter-preserving surgery offers significant functional advantages over APR, it is frequently associated with LARS, one of the most common postoperative complications of rectal cancer (9). LARS is characterized by intestinal dysfunction, with symptoms that may persist for years after surgery, significantly limiting patients’ quality of life and causing considerable psychological distress. Existing treatment strategies are either limited in efficacy, invasive, or costly (10). Therefore, developing safe and effective rehabilitation protocols for LARS represents a highly meaningful clinical challenge.

Traditional Chinese Medicine (TCM) is increasingly being incorporated into cancer diagnosis and treatment systems due to its ability to improve treatment tolerability, alleviate symptoms, and enhance quality of life. However, evidence supporting its efficacy in promoting resection therapy for ultra-low rectal cancers or improving postoperative intestinal dysfunction remains limited.

Here, we report a case of a patient with KRAS gene mutation in an extremely low-grade locally advanced rectal adenocarcinoma: despite receiving multiple lines of radiotherapy, chemotherapy, and immunotherapy without achieving adequate tumor regression, the patient successfully underwent sphincter-preserving radical surgery under a combined traditional Chinese and Western medicine treatment regimen. We also report the evolution of postoperative major LARS during multimodal rehabilitation centered on electro acupuncture at the Baliao points. The entire treatment process was meticulously documented in accordance with the CARE Case Report Guidelines and the STRICTA Acupuncture Clinical Trial Intervention Reporting Standards, aiming to provide clinical evidence for integrated treatment strategies tailored to this patient population to optimize both oncological outcomes and functional recovery (11–13).

## Case report

### Patient information

A 55-year-old female presented in April 2022 with “hematochezia accompanied by prolapse of a perianal mass for over one year.” One year prior, the patient developed hematochezia without obvious precipitating factors, characterized by bright red blood (approximately 5–10 mL) adhering to the stool surface, accompanied by anal tenesmus and prolapse of a perianal mass during defecation; these symptoms were recurrent and progressively worsening. The patient had undergone total hysterectomy due to gynecological cysts in 2017. She reported no smoking, alcohol use, or family history of malignancy. The potential need for a permanent stoma caused substantial distress, and sphincter preservation was her stated treatment priority.

### Clinical findings and diagnostic assessment

**Figure 1:**
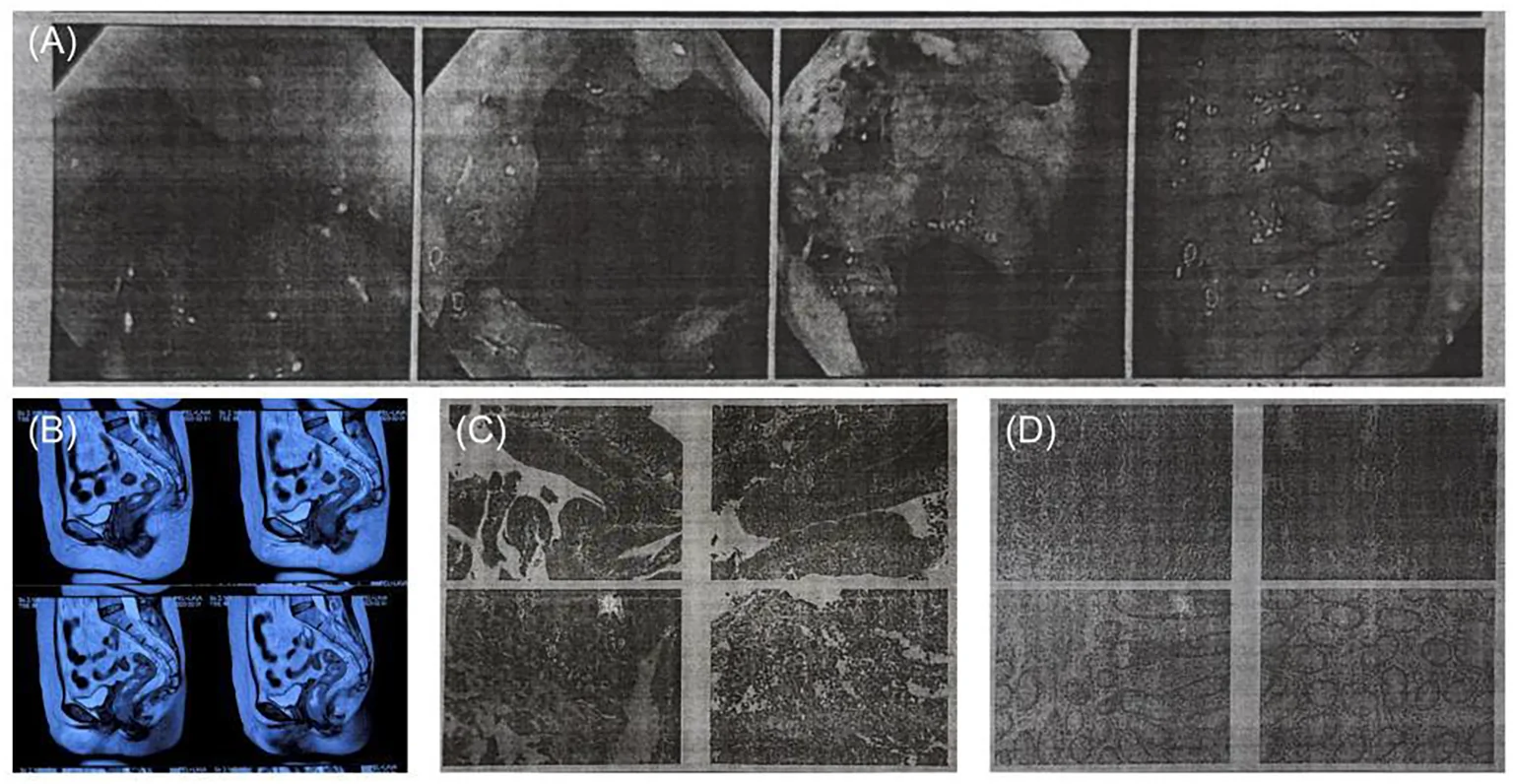
In 2023, the patient underwent examinations and was diagnosed with ultra-low rectal adenocarcinoma (cT3aN1M0, IIIB). **(A)** Colonoscopy revealed an ulcerative lesion in the anal canal and the posterior wall of the distal rectum, with invasion extending approximately two-thirds of the anal canal length. **(B)** Pelvic sagittal contrast-enhanced MRI demonstrated that the tumor was located in the mid-to-lower rectal segment, spanning 5.7 cm across the peritoneal reflection; the inferior border of the tumor was situated 4 mm distal to the inferior margin of the anus. **(C, D)** Pathological examination of the colonoscopic biopsy specimen showed: moderate to severe atypical hyperplasia with focal carcinoma (adenocarcinoma) in the rectal glandular epithelium; chronic inflammation accompanied by polypoid hyperplasia was observed in the sigmoid colon mucosa; mild atypical hyperplasia was also noted in the local glandular epithelium.

Physical examination revealed, during a digital rectal examination, a fragile, easily bleeding irregular ulcerative mass palpable in the lower rectum. An anorectal endoscopy demonstrated an irregular ulcer on the posterior wall of the anal canal and distal rectum, with its circumferential extension covering approximately two-thirds of the rectal surface. The ulcer surface was covered with an ulcerative coating, the margins were raised and ridge-like, the texture was friable, and it exhibited significant bleeding upon palpation (**Figure 1A**).

To establish a definitive diagnosis and staging, the patient underwent comprehensive systemic examinations at an external hospital from January to February 2023. High-resolution pelvic MRI evaluation (**Figure 1B**) revealed a low rectal carcinoma with a tumor extending approximately 5.7 cm along the longitudinal direction of the bowel, penetrating the serosal layer and involving the anal canal. Notably, the lower border of the tumor was only 4 mm from the anal margin, indicating an ultra-low rectal carcinoma, and invasion of the internal anal sphincter should be ruled out. Additionally, several small lymph nodes with diameters <5 mm were observed within the mesorectum, with the shortest distance between the tumor and the mesorectal fascia <0.1 cm, demonstrating a circumferential positive margin and a high-risk status; the score for extramural vascular invasion was 2. Based on these imaging findings, the initial staging was cT3aN1M0, corresponding to stage IIIB. Colonoscopy confirmed these findings, and concurrent cryoexcision of multiple colonic polyps was performed at a distance of 25 cm from the anus. Pathological biopsy confirmed rectal adenocarcinoma, while sigmoid colon polyps exhibited chronic mucosal inflammation with mild atypical hyperplasia (**Figures 1C, D**). Subsequent genetic testing revealed molecular characteristics including KRAS mutation (variant level I), a tumor mutation burden of 15.41 Muts/Mb, and MSI-L/MSS status.

**Figure 2:**
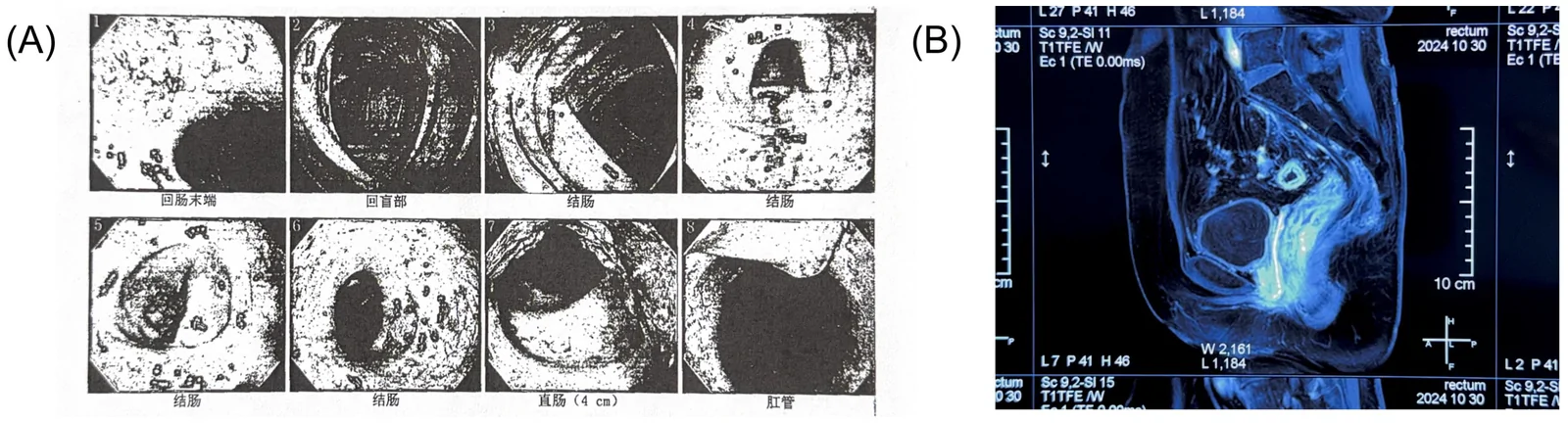
The patient underwent a series of treatments at an external hospital and was subsequently evaluated for their condition. **(A)** Colonoscopy revealed an ulcerated mass located in the rectum approximately 4 cm from the anal verge; the base of the ulcer was deep and covered with copious fecal matter, the ulcer margin exhibited irregular elevation, the mass had a friable texture that caused bleeding upon palpation, and the intestinal lumen at the site of the mass demonstrated eccentric stenosis. **(B)** MRI imaging showed that the size of the rectal mass had decreased from the initial measurement of 5.7 cm to 3.6 cm, but the mass remained present.

The patient initiated systematic treatment at an external hospital starting in February 2023. Initially, they received six cycles of chemotherapy (XELOX and mFOLFOX6 regimens), followed by pelvic radiotherapy combined with capecitabine chemotherapy in July 2023. During the planning of the 8th cycle of chemotherapy in October 2023, severe adverse reactions to oxaliplatin (including dyspnea, profuse sweating, and chills) occurred. After symptomatic relief was achieved, treatment was discontinued. Subsequently, during immunotherapy with capecitabine in combination with sintilimab, severe intolerable adverse reactions—including somnolence, fatigue, cutaneous hyperpigmentation, palm macules, and palpitations—occurred, ultimately leading to the discontinuation of immunotherapy. Following this, the patient received intermittent monotherapy with capecitabine combined with traditional Chinese medicine outside the hospital. In October 2024, the patient returned for further clinical evaluation. A comprehensive assessment revealed that although the size of the rectal mass had reduced from the initial measurement of 5.7 cm to 3.6 cm, it remained present (**Figures 2A, B**). The pathological findings indicated poorly differentiated adenocarcinoma with partial neuroendocrine differentiation, a Ki-67 index as high as 90%, and a poor biological behavior. The multidisciplinary team concluded that, despite the implementation of various treatment modalities, the degree of tumor regression was insufficient to achieve the safe distal surgical margin required for proctectomy.

### Therapeutic intervention

We have developed a translational treatment regimen integrating traditional Chinese and Western medicine with multi-target synergy. For Western medical therapy, we adopted a regimen combining bevacizumab with irinotecan and capecitabine. The specific regimen was as follows: bevacizumab 400 mg daily on day 1; irinotecan 300 mg daily on day 1; and capecitabine 1.0 g morning and 1.0 g evening from day 1 to day 14, administered in cycles of 21 days for a total of 5 cycles. The subsequent dosage regimen was adjusted based on patient tolerance. Postoperative adjuvant chemotherapy employed regimens such as FOLFIRI or capecitabine monotherapy. Traditional Chinese therapies focused on two key aspects: modulating the microenvironment of local lesions and reshaping systemic immune function. All procedures were performed by physicians with over two years of clinical experience in integrated traditional Chinese and Western medicine, strictly adhering to aseptic techniques, with moderate individualized adjustments made according to changes in the patient’s clinical manifestations during each visit.

At the local treatment level, fire needle puncture was performed once before and once after chemotherapy on all eight Liao acupoints (bilaterally: Shangliao BL31, Ciliao BL32, Zhongliao BL33, Xialiao BL34). A 0.40 × 25 mm tungsten steel fire needle was used; after heating it to a white color over an alcohol lamp, it was rapidly inserted into each acupoint at a depth of approximately 0.5 cm with a single puncture per point. The needle was immediately removed without leaving any residue.

Pricking bloodletting combined with cupping was performed once weekly over palpable reactive areas in the lumbosacral region. Before each session, the lumbosacral area was systematically palpated to identify localized nodules, cord-like indurations, or areas of increased tenderness. After routine skin disinfection, the selected reactive area was superficially pricked 5–8 times using a sterile, single-use bloodletting needle, with an approximate penetration depth of 2–3 mm. A sterile cup was then immediately applied over the treated area under negative pressure and retained for 10–15 min to facilitate local bloodletting. Approximately 3–5 mL of blood was released during each session. After removal of the cup, the treatment area was cleaned and compressed with sterile gauze until complete hemostasis was achieved.

For refractory nodules in the abdominal and lumbar regions, administer weekly localized release therapy with a blade needle combined with composite manual manipulation: first relax muscles via kneading, then apply plucking techniques to adhesions; subsequently use a 0.50 mm blade needle for release followed by pressure application to achieve hemostasis and further dissipate tissue aggregation.

Additionally, sublingual bloodletting therapy is performed once every two weeks: using a disposable lancet to rapidly puncture the Jinjin (EX-HN12) and Yuye (EX-HN13) acupoints to release approximately 1–2 mL of blood, followed by rinsing the mouth with warm water to promote blood circulation and resolve stasis.

At the systemic regulation level, routine acupuncture therapy is administered three times weekly. The primary acupoints selected include both Shenshu (BL23), Mingmen (GV4), Xuehai (SP10), Sanyinjiao (SP6), Neiguan (PC6), Fenglong (ST40), Gongsun (SP4), Taibai (SP3), Yinlingquan (SP9), Qimen (LR14), and Waiguan (SJ5). Disposable sterile acupuncture needles (0.30 × 40 mm, Hwato) are used, with standard insertion depths of approximately 15–25 mm. The technique involves applying balanced tonifying and draining maneuvers until the practitioner feels firmness and the patient reports a sensation of soreness, numbness, distension, or heaviness; each session involves needle retention for 30 minutes.

Moxibustion and finger-acupuncture therapy are on each day of chemotherapy and the following day. Smoke-free moxa sticks are applied via circular moxibustion at the Shenshu, Mingmen, and Baliao acupoints for 5–10 minutes per point, inducing local skin erythema and a sense of warmth. During breaks in moxibustion, the thumb pad is used to apply continuous pressure to these acupoints for about 1 minute each. Rotational moxibustion is applied gently to Zhongwan (RN12), Qihai (RN6), and Guanyuan (RN4) for 20 minutes per acupoint to warm and replenish primordial qi, promote the generation of defensive yang, and reduce the risk of bone marrow suppression post-chemotherapy.

All detailed information regarding TCM treatments is presented in **Table 1**.

**Table 1:** Detailed protocol of traditional Chinese medicine interventions administered during integrated conversion treatment.

Intervention	Treatment sites	Equipment	Parameters	Frequency and timing
Fire-needle acupuncture	Bilateral Baliao acupoints: Shangliao (BL31), Ciliao (BL32), Zhongliao (BL33), and Xialiao (BL34)	Tungsten-steel fire needle, 0.40 × 25 mm	One insertion per acupoint; insertion depth approximately 5 mm	Once before and once after each chemotherapy cycle
Pricking bloodletting combined with cupping	Palpable reactive areas in the lumbosacral region, identified as localized nodules, cord-like indurations, or areas of increased tenderness	Sterile single-use bloodletting needle and sterile cupping device	5–8 superficial punctures; depth 2–3 mm; cupping retained for 10–15 min; approximately 3–5 mL of blood released per session	Once weekly
Blade-needle release with manual manipulation	Persistent palpable nodules or cord-like soft-tissue indurations in the abdominal and lumbar regions	Sterile single-use blade needle, approximately 0.50 mm*	Localized release according to the size and location of the palpable lesion; no needle retention	Once weekly for persistent/refractory reactive areas
Sublingual pricking bloodletting	Bilateral Jinjin (EX-HN12) and Yuye (EX-HN13)	Sterile disposable lancet	Approximately 1–2 mL total blood released per session	Once every 2 weeks
Manual acupuncture	Shenshu (BL23), Mingmen (GV4), Xuehai (SP10), Sanyinjiao (SP6), Neiguan (PC6), Fenglong (ST40), Gongsun (SP4), Taibai (SP3), Yinlingquan (SP9), Qimen (LR14), and Waiguan (SJ5), as clinically indicated	Sterile disposable acupuncture needles, 0.30 × 40 mm (Hwato)	Insertion depth approximately 15–25 mm; needle retention for 30 min	Three sessions per week
Moxibustion	Shenshu (BL23), Mingmen (GV4), and bilateral Baliao acupoints (BL31–BL34), Zhongwan (CV12), Qihai (CV6), and Guanyuan (CV4)	Moxa sticks*	5–10 min per acupoint. Abdominal approximately 20 min per acupoint	On the day of chemotherapy and the following day
Finger acupuncture	Shenshu (BL23), Mingmen (GV4), and bilateral Baliao acupoints (BL31–BL34)	Thumb-pad pressure; no additional device	Approximately 1 min per acupoint	Performed together with moxibustion on the day of chemotherapy and the following day

Herbal medicine was prescribed according to syndrome differentiation based on modified Banxia Xiexin Decoction combined with Fuyuan Huoxue Decoction. The detailed herbal composition is provided in **Table 2**.

**Table 2:** Composition of the modified Banxia Xiexin Decoction combined with Fuyuan Huoxue Decoction.

Herb	Latin name	Dose (g)	Traditional indication	Reported pharmacological activity
Pinellia ternata	*Pinelliae Rhizoma*	10	Resolve phlegm	Anti-inflammatory, gastrointestinal protection
Coptis root	*Coptidis Rhizoma*	5	Clear heat	Berberine-mediated anti-tumor and anti-inflammatory effects
Baical skullcap root	*Scutellariae Radix*	10	Clear heat	Anti-inflammatory, anti-proliferative
Rhubarb root	*Rhei Radix et Rhizoma*	10	Remove blood stasis	Immunomodulatory, anti-tumor
Chinese Angelica	*Angelicae Sinensis Radix*	15	Nourish blood	Anti-fibrotic, immunomodulatory
Safflower	*Carthami Flos*	10	Promote circulation	Anti-angiogenic, microcirculation improvement
Leech	*Hirudo*	5	Break blood stasis	Anticoagulant, improves microcirculation
Mongolian Snakegourd Root	*Trichosanthis Radix*	15	Dissipate masses	Anti-inflammatory, immune regulation
Twotooth Achyranthes Root	*Achyranthis Bidentatae Radix*	15	Promote circulation	Neuroprotective, anti-inflammatory
Liquorice root	*Glycyrrhizae Radix et Rhizoma*	6	Harmonize prescription	Anti-inflammatory, hepatoprotective

The herbal prescription was individualized according to the patient’s clinical manifestations during treatment while maintaining the core composition listed above.

The proposed therapeutic rationale is based on traditional Chinese medicine theory and is provided for descriptive purposes only; it should not be interpreted as an established pharmacological mechanism.

**Figure 3:**
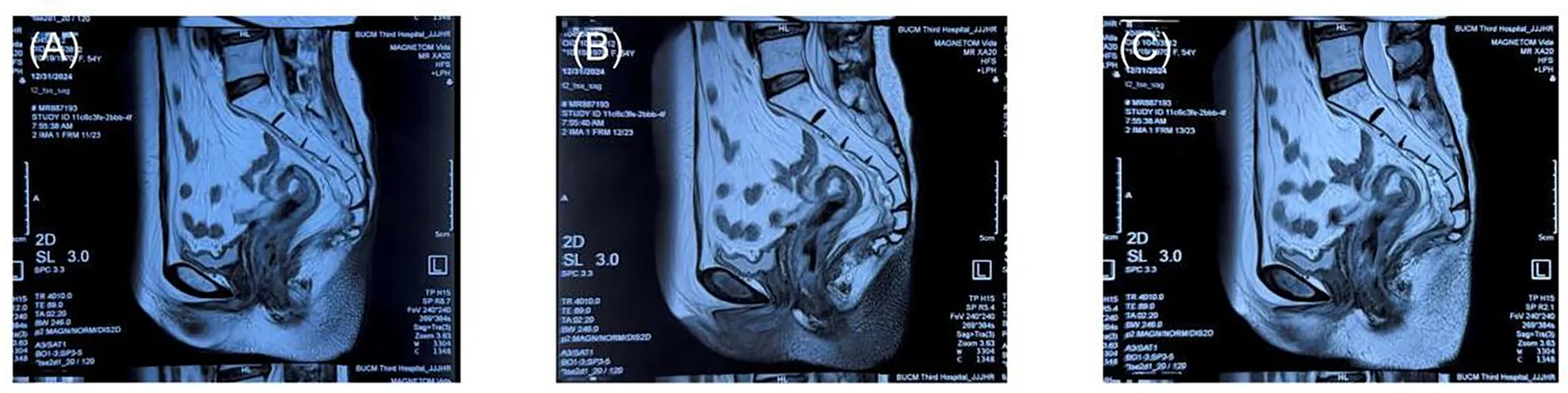
The patient underwent evaluation after completing two cycles of treatment with bevacizumab combined with irinotecan and capecitabine. **(A–C)** High-resolution pelvic MRI demonstrated that, compared with previous measurements, the degree of rectal wall thickening was significantly reduced, with the maximum thickness measuring 10 mm.

After two treatment cycles with the aforementioned regimen, the disease status was re-evaluated as stable. High-resolution pelvic MRI revealed significant reduction in rectal wall thickening compared to prior measurements (the thickest area measured at MR on December 31, 2024, was approximately 10 mm, indicating a decrease). The surgical margins were improved, creating favorable conditions for surgery (**Figures 3A–C**).

### Surgical treatment and postoperative pathology

After completion of five cycles of conversion treatment, the patient underwent laparoscopic radical rectal resection with transanal specimen extraction using a Bacon-type pull-through procedure on June 23, 2025. Intraoperative exploration revealed no ascites, peritoneal metastatic nodules, or visible abnormalities of the stomach, spleen, or pancreas. The tumor was located in the lower rectum, approximately 2.5 cm from the anal verge, without macroscopic serosal involvement. This measurement was obtained after treatment and differs in timing and method from the pretreatment MRI distance of approximately 4 mm.

The rectum and mesorectum were mobilized laparoscopically according to the principles of total mesorectal excision, with preservation of the hypogastric nerve plexuses, left ureter, gonadal vessels, and left colic artery. Lymphatic and adipose tissues around the inferior mesenteric vessels were dissected, and bilateral lateral pelvic dissection with clearance of visible lymphatic tissue was performed. Near the anal verge, dissection entered the intersphincteric plane between the internal and external anal sphincters. However, the operative record did not document formal partial, subtotal, or total intersphincteric resection.

The proximal bowel was divided approximately 10 cm above the upper tumor margin. Distally, the anal canal was opened near the dentate line approximately 1 cm below the tumor, allowing complete transanal specimen extraction. The proximal colon was exteriorized approximately 5 cm through the anus and secured to the anal-canal mucosa with interrupted sutures, consistent with a Bacon-type pull-through reconstruction. No suture anastomosis or diversion stoma creation was performed. Estimated blood loss was approximately 20 mL, and no intraoperative transfusion or complication occurred. Subsequent colonoscopy identified the reconstructed anastomotic site approximately 1.0 cm from the anal verge.

Histopathological examination demonstrated moderately to poorly differentiated ulceroinfiltrative rectal adenocarcinoma invading the deep muscularis propria and involving the dentate-line region, with focal extension into the anal-canal squamous epithelium. Treatment-related tumor-cell degeneration, necrosis, inflammatory infiltration, and fibrous proliferation were consistent with Dworak tumor regression grade 2. Perineural invasion was identified, whereas no definite lymphovascular tumor embolus was observed. The proximal, distal, and circumferential resection margins were negative, supporting an R0 resection. The separately submitted anococcygeal ligament specimen showed no tumor involvement. None of the 12 examined lymph nodes contained metastatic carcinoma, resulting in a pathological stage of ypT2N0. Immunohistochemistry demonstrated BRAF V600E negativity, C-MET expression of 1+, HER2 score 0, and preserved expression of MLH1, MSH2, MSH6, and PMS2.

### Rehabilitation therapy and follow-up

**Figure 4:**
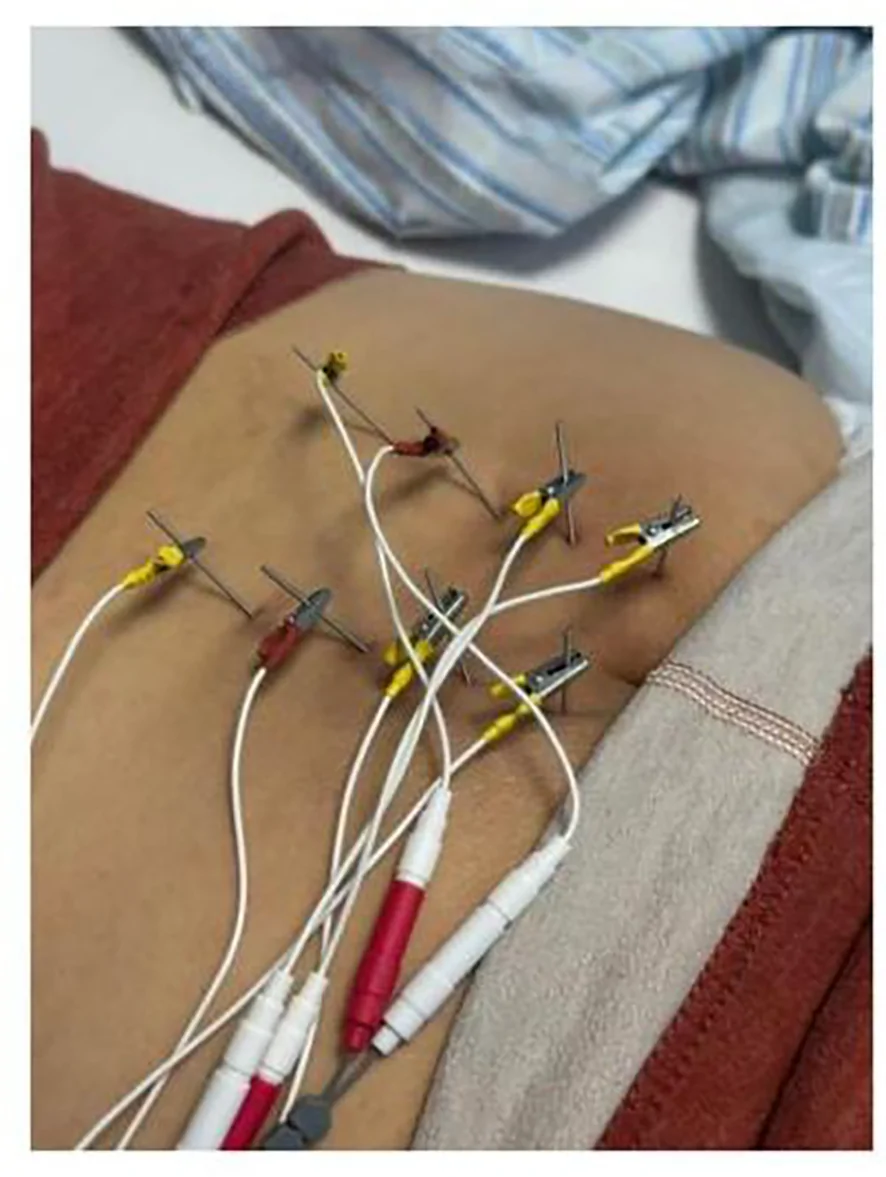
Electroacupuncture administered at the bilateral Baliao acupoints (BL31–BL34) in the lumbosacral region.

Less than one month postoperatively, the patient developed major LARS, characterized by up to 25–30 daily bowel movements accompanied by severe anal tenesmus, resulting in an extremely poor quality of life. Starting from August 2025, the patient began receiving acupuncture treatment at Baliao points—specifically, the eight points comprising Shangliao (BL31), Ciliao (BL32), Zhongliao (BL33), and Xialiao (BL34), bilaterally. Following skin disinfection, the needles were inserted. After insertion, manual stimulation techniques, including lifting, thrusting, and rotating, were applied to elicit the deqi sensation, which was a composite feeling of soreness, numbness, distension, or heaviness. The needling depths were approximately 75 mm (3 Cun). Electroacupuncture parameters were set to a continuous wave at a frequency of 50 Hz and a current intensity of 1–5 mA for 30 minutes per session (14). EA is illustrated in **Figure 4**. Patients were treated three times a week for four consecutive weeks, 12 sessions in total. This treatment significantly improved the patient’s bowel movement issues. After 4 weeks of therapy, the daily frequency of bowel movements decreased to 7–10 times, the sensation of anal fullness virtually disappeared, and both appetite, sleep quality, and body weight showed improvement. Regular follow-up at 1, 3, and 6 months demonstrated stable outcomes with continued improvement.

To objectively evaluate the therapeutic response, the Chinese-validated version of the LARS score (total range 0–42, categorized as no LARS [0–20], minor LARS [21–29], and major LARS [30–42]) was administered before treatment and after every three treatment sessions. In addition, the MSKCC-BFI questionnaire – comprising 18 items across three subscales and the EORTC QLQ-C30 questionnaire were completed before treatment and after the 12-session course.

Following treatment, the patient exhibited marked improvement in the LARS score, decreasing from 41(major LARS) to 29(minor LARS) points. Although the LARS scale indicates that the patient still meets the criteria for LARS, symptoms have significantly improved, with reduced urgency and frequency of bowel movements, as shown in **Table 3**.

**Table 3:** LARS score before and after treatment.

Question	2025-09-01 (baseline)	2025-09-07 (3 EA)	2025-09-13 (6 EA)	2025-09-19 (9 EA)	2025-09-27 (12 EA)	2025-10-27 (1month)	2025-12-28 (3months)	2026-03-29 (6months)
Total Score								
	41	41	36	36	29	29	22	11
Do you ever have occasions when you cannot control your flatus(wind)?								
	7	7	4	4	4	4	0	0
Do you ever have any accidental leakage of liquid stool?								
	3	3	3	3	3	3	0	0
How often do you open your bowels?								
	4	4	4	4	2	2	2	2
Do you ever have to open your bowels again within one hour of the last bowel opening?								
	11	11	9	9	9	9	9	9
Do you ever have such a strong urge to open your bowels that you have to rush to the toilet?								
	16	16	16	16	11	11	11	0

The MSKCC-BFI score showed an improvement from 39 to 54 after treatment, and the patient’s average daily bowel movements decreased from 30 to 7. The therapeutic effect was primarily manifested in alleviating frequent and urgent bowel movements, as shown in **Table 4**.

**Table 4:** MSKCC-BFI score before and after treatment.

Dimension	Baseline	Following treatment
Total Score	39	54
Urgent Stool Frequency Factor	21	31
Diarrhea influenced by dietary factors	10	10
Abnormal Defecation Sensations Factor	8	13
Average daily bowel movements	30	7

The EORTC QLQ-C30 questionnaire indicates that patients experienced improvements in social functioning following treatment. Pain improved but did not completely resolve, and global health increased from 50 to 75, as shown in **Table 5**.

**Table 5:** EORTC QLQ-C30 score before and after treatment.

Dimension	Baseline	Following treatment
Physical functioning	100	100
Role functioning	66.67	66.67
Emotional functioning	100	100
Cognitive functioning	83.33	83.33
Social functioning	83.33	100
Fatigue	0	11.11
Nausea and vomiting	0	0
Pain	50	16.67
Dyspnea	0	0
Insomnia	0	0
Appetite loss	0	0
Constipation	0	0
Diarrhea	0	0
Financial difficulties	33.33	33.33
Global Health	50	75

**Figure 5:**
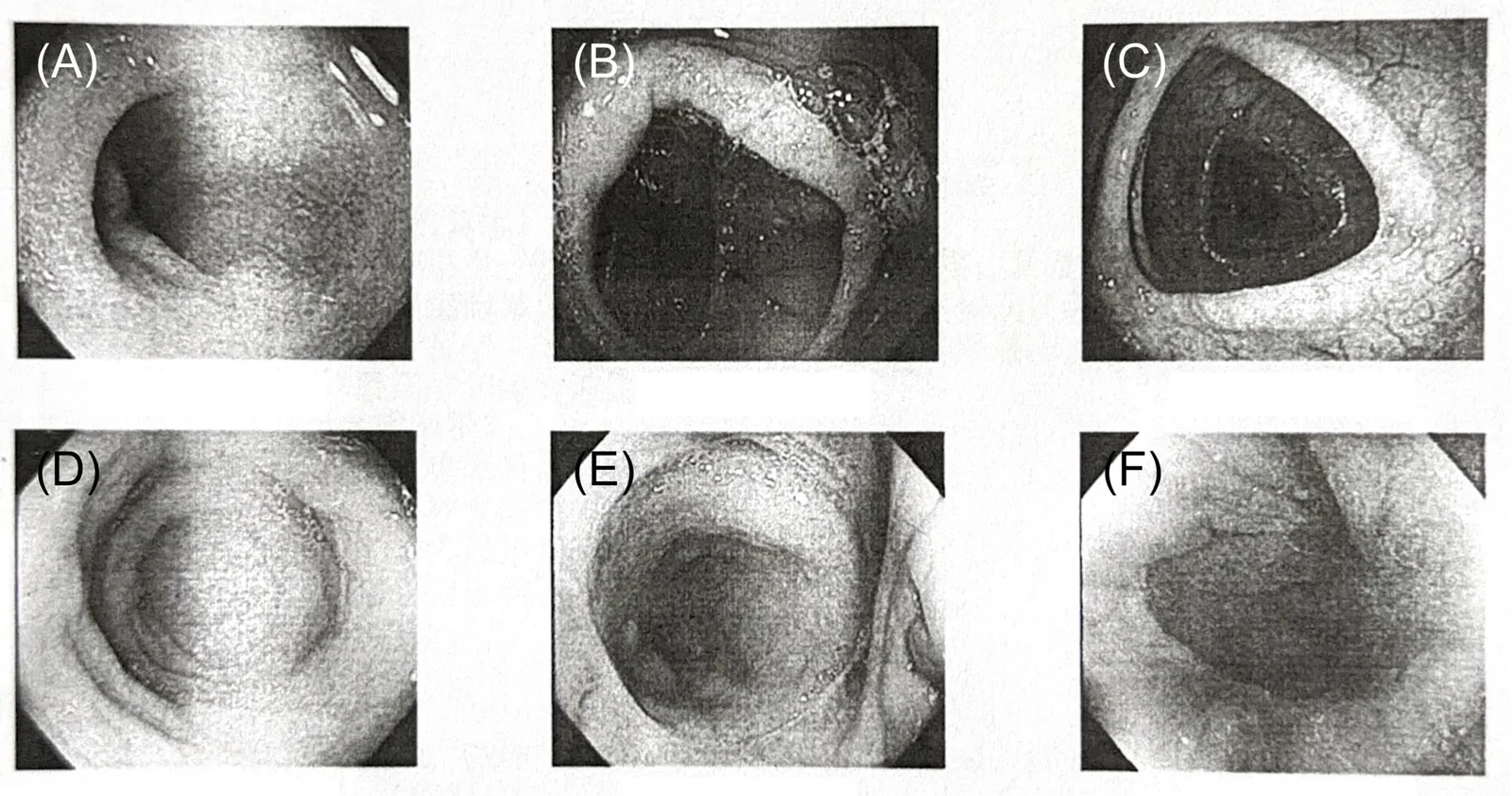
Following the Bacon procedure for rectal cancer, follow-up colonoscopy was performed. **(A–F)** Representative colonoscopic views showed a patent intestinal lumen and no significant abnormality or stenosis at the anastomotic site.

At the final follow-up visit (May 28,2026), the patient’s condition remained stable following maintenance therapy with capecitabine monotherapy combined with traditional Chinese medicine rehabilitation therapy; a repeat colonoscopy showed no signs of recurrence or metastasis; a anastomotic site approximately 1.0 cm from the rectal-anal margin was observed, which appeared smooth without any significant stenosis (**Figure 5**). Tumor marker levels remained within normal ranges (CA19-9: 15.30 U/mL, CEA: 3.41 ng/mL).

**Figure 6:**
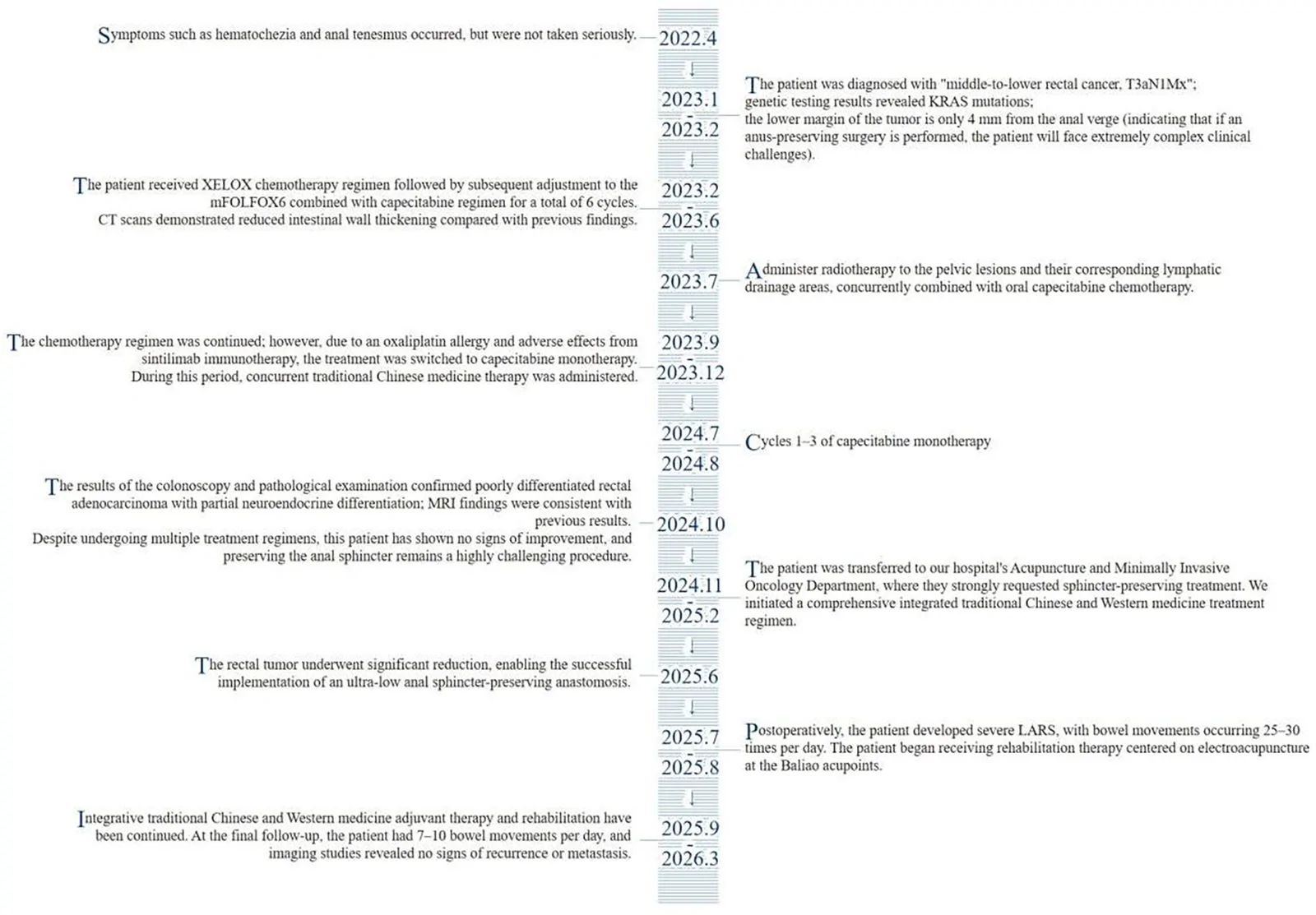
Timeline of diagnosis and treatment process.

The timeline of all key nodes in the treatment process is shown in **Figure 6**.

## Discussion

This case describes a patient with KRAS-mutant ultra-low rectal cancer initially staged as cT3aN1M0, with a tumor margin 4 mm from the anal verge, mesorectal fascia involvement, imaging findings indicated positive circumferential resection margins (MRF involvement) and an extramural vascular invasion (EMVI) score of 2. Following an inadequate response to prior chemotherapy, radiotherapy, and immunotherapy, she received five cycles of bevacizumab–XELIRI alongside traditional Chinese medicine interventions. Subsequent laparoscopic sphincter-preserving resection with Bacon-type pull-through reconstruction achieved R0 resection and ypT2N0 pathology. Postoperative major LARS improved during multimodal rehabilitation incorporating electroacupuncture, with bowel frequency decreasing from 25–30 to 7–10 movements daily. As a descriptive single-case report, these observations demonstrate temporal associations rather than causality; neither tumor downstaging nor functional improvement can be attributed to any individual therapeutic component.

Preserving the anus in ultra-low rectal cancers (where the tumor margin is ≤5 cm from the anal verge, with only 4 mm in this case) remains one of the most challenging issues in colorectal surgery. For patients with tumors extremely close to the anal verge who initially require abdominoperineal resection (APR), whether neoadjuvant therapy can be converted into an anus-preserving procedure reflects the efficacy of the conversion strategy. The Greccar 1 trial is a pivotal Phase III study designed to evaluate the feasibility of anal preservation after neoadjuvant therapy in patients with ultra-low-risk rectal cancer; its 10-year follow-up results demonstrated an overall anus-preserving resection rate of 85%, including 72% for intersphincteric resection (ISR) (9). Additionally, neoadjuvant therapy followed by TME is recommended for locally advanced/low rectal cancer, particularly when CRM is threatened, as it promotes tumor downstaging and facilitates R0/CRM-negative resection (15).

However, for patients with KRAS mutation-positive tumors, conventional chemoradiotherapy fails to achieve the desired therapeutic efficacy. KRAS is one of the most common mutated genes in colorectal cancer, present in approximately 40% of patients (16). KRAS-mutated colorectal cancers not only exhibit resistance to targeted anti-EGFR therapies but also demonstrate reduced sensitivity to conventional chemotherapy and radiotherapy, which is associated with poorer prognosis and shorter disease-free survival. A meta-analysis incorporating 21 studies demonstrated that patients with KRAS-mutated locally advanced rectal cancer who received neoadjuvant chemoradiotherapy had significantly lower probabilities of achieving favorable treatment responses compared to those with wild-type KRAS (OR = 0.52,95% CI: 0.31–0.86, p = 0.01) and lower rates of pathological complete response (OR = 0.73,95% CI: 0.55–0.97, p = 0.02) (17). In this case, poor response to prior multiple-line standard therapy was primarily attributed to KRAS gene mutation. Thus, the most prominent feature of this case lies in the dual challenges of “refractory tumor biology” and “poor treatment tolerance.”

The difference in surgical approach is crucial. This patient underwent laparoscopic TME combined with transanal specimen removal and Bacon-type manual suturing and extraction reconstruction, rather than the traditional stapled low anterior resection. The procedure included total mesorectal excision, anatomical dissection within the intersphincteric plane, distal transverse resection near the dentate line, and transanal extrusion of the proximal colon, which was then secured to the anal canal mucosa using interrupted sutures. A recent meta-analysis of ISR procedures demonstrated that severe LARS occurred in 46% of patients (95% confidence interval: 33%–60%), with significant heterogeneity across the studies (18, 19). Another contemporary cohort study involving 128 patients who underwent manual suturing ISR combined with protective ileostomy reported that 61 patients (47.7%) developed severe LARS following stoma reduction (20). However, unlike the patients in the aforementioned cohort study, this patient did not undergo ileostomy. Therefore, when managing subsequent major LARS, a comprehensive assessment based on the surgical approach and the patient’s preoperative medical history should be conducted, rather than attributing the condition to a single factor. Compared with conventional neoadjuvant regimens, this conversion therapy case considered the patient’s KRAS mutation status and prior oxaliplatin allergy history, leading to the selection of the bevacizumab–irinotecan + capecitabine (XELIRI) regimen for chemotherapy. A multicenter phase II study assigned patients with KRAS-mutated locally advanced rectal cancer to mFOLFOX6 plus bevacizumab and reported favorable resection outcomes across the overall cohort (21). That study used an oxaliplatin-based regimen and cannot establish the efficacy of bevacizumab–XELIRI or justify causal attribution in the present patient. Likewise, evidence regarding KRAS and neoadjuvant response is mixed; attributing prior treatment failure solely to KRAS mutation would overstate the available evidence.

The innovation of this therapeutic approach lies in integrating traditional Chinese medical techniques—such as fire needle acupuncture targeting the Baliao points, bloodletting cupping therapy, blade needle release, bloodletting at Jinjin (EX-HN12) and Yuye (EX-HN13), acupuncture, moxibustion, and oral administration of traditional Chinese medicine—as synergistic treatment modalities, rather than relying solely on Western-style symptomatic management. However, these simultaneously implemented TCM interventions represent synergistic measures grounded in TCM principles; there is no specific evidence indicating that they exert direct effects such as tumor hyperthermia, vascular disruption, immune activation, or synergistic antitumor efficacy. In 2019, our team reported a case of a 38-year-old male patient with ultra-low rectal cancer (cT4N0–2M0, Stage III), in whom no significant tumor regression was observed after 6 cycles of neoadjuvant chemotherapy; however, following additional treatment with Baliao fire needles combined with another 6 cycles of therapy, imaging studies demonstrated complete resolution of the tumor, and postoperative pathology revealed no residual tumor cells, achieving ypT0N0M0 pathological complete remission (22). In contrast to this case, although the tumor regression grade was only Dworak TRG 2 (mild treatment response), given the patient’s KRAS mutation status and history of multi-line drug resistance, renders it of significant diagnostic relevance. LARS is the most common functional complication following sphincter-preserving surgery for rectal cancer, with an incidence rate as high as 80%–90% (23), among which major LARS accounts for 64.58% (24). In this case, patients experienced 25–30 daily bowel movements postoperatively; the baseline LARS score was 41 points, indicating major LARS. Multivariate logistic regression analysis identified obesity (BMI ≥25 kg/m²), neoadjuvant radiotherapy (OR = 5.181), an anastomosis-to-anal margin distance <5 cm (OR = 4.600), and postoperative anastomotic leakage (OR = 4.963) as independent risk factors for major LARS; furthermore, an anastomosis-to-anal margin distance ≤4 cm was strongly associated with a higher incidence of LARS (p<0.001) (25). In this case, the patient had undergone pelvic radiotherapy prior to surgery, with the anastomosis located at an extremely low position, indicating high-risk characteristics. Furthermore, several studies have shown that LARS symptoms often reach a plateau approximately 4 years postoperatively, suggesting that symptoms may persist for an extended period in some patients, necessitating effective intervention measures (26). Currently, modern treatment options are limited, primarily comprising pelvic floor rehabilitation exercises, transanal irrigation, and sacral nerve stimulation therapy; however, these methods often demonstrate suboptimal efficacy, entail high costs, and involve invasive procedures, making them difficult to widely adopt (27).

Electroacupuncture therapy exhibits a time-dependent correlation with the reduction in defecation frequency as well as improvements in the LARS score, intestinal function, and certain quality-of-life scores. Previously, our research team reported on the therapeutic efficacy observed during extended electroacupuncture treatment at the Baliao acupoint in two patients; however, since this study employed a non-controlled design, no definitive conclusions regarding its therapeutic efficacy could be drawn (28). From an anatomical perspective, the Baliao acupoint is located at the posterior sacral foramen, and its deep region precisely corresponds to the pathway taken by the sacral nerve roots emerging from the sacral canal, exhibiting significant overlap with the S2–S4 sacral nerves that innervate the rectum, anal canal, and pelvic floor muscles.

The limitations of this case report include its single-patient design; absence of a control group, blinding, and objective anorectal assessment; concurrent administration of chemotherapy, herbal therapy, and supportive rehabilitation; potential expectation effects; and incomplete documentation of the bevacizumab washout interval and pull-through staging. Given that postoperative LARS symptoms typically improve gradually, the observed reduction in the LARS score—from 29 points to 11 points after 6 months—may be attributable to factors such as natural recovery, physiological adaptation, dietary adjustments, the combination treatment regimen, or the timing of intervention postoperatively. Therefore, the findings of this study should be regarded as descriptive data and as a basis for formulating hypotheses; future prospective controlled trials are warranted to validate these findings.

## Patient perspective

When I was told that the tumor was too close to the anus and that I would most likely need a permanent colostomy, I felt devastated. Preserving my anus was my greatest wish. After being referred to this hospital, my doctors did not give up on me. They developed an individualized treatment plan that combined chemotherapy with traditional Chinese medicine, including acupuncture. Throughout the treatment, I gradually felt stronger and noticed that my symptoms were improving. The happiest moment was when I was told that sphincter-preserving surgery had been successfully performed.

Although I developed severe bowel dysfunction after the operation and had to use the toilet more than 20 times a day, electroacupuncture at the Baliao acupoints brought remarkable improvement. My bowel frequency gradually decreased, the feeling of urgency almost disappeared, and I was finally able to return to a more normal daily life. I am deeply grateful to my medical team for helping me preserve both my life and my dignity.

## Conclusion

In this patient with KRAS-mutated ultra-low rectal cancer, an individualized conversion-treatment course was followed by R0 sphincter-preserving resection. Major LARS subsequently improved over six months during multimodal rehabilitation centered on electroacupuncture. The case supports feasibility and a temporal association with functional improvement, but it does not establish the effectiveness of either the combined oncological strategy or electroacupuncture. These observations are hypothesis-generating and should be evaluated in controlled studies.

## Data Availability

The original contributions presented in the study are included in the article/**Supplementary Material**. Further inquiries can be directed to the corresponding authors.
